# Activation of the STAT3 Signaling Pathway by the RNA-Dependent RNA Polymerase Protein of Arenavirus

**DOI:** 10.3390/v13060976

**Published:** 2021-05-25

**Authors:** Qingxing Wang, Qilin Xin, Weijuan Shang, Weiwei Wan, Gengfu Xiao, Lei-Ke Zhang

**Affiliations:** 1 State Key Laboratory of Virology, Wuhan Institute of Virology, Chinese Academy of Sciences, Wuhan 430071, Hubei, China; wangqingxing16@mails.ucas.ac.cn (Q.W.); shangweijuan@wh.iov.cn (W.S.); weiwei-wan@outlook.com (W.W.); 2 University of Chinese Academy of Sciences, Beijing 100049, China; 3 UMR754, Viral Infections and Comparative Pathology, 50 Avenue Tony Garnier, CEDEX 07, 69366 Lyon, France; colinxin1993@outlook.com

**Keywords:** arenavirus, LCMV, STAT3, RNA-dependent RNA polymerase, virus–host interactions

## Abstract

Arenaviruses cause chronic and asymptomatic infections in their natural host, rodents, and several arenaviruses cause severe hemorrhagic fever that has a high mortality in infected humans, seriously threatening public health. There are currently no FDA-licensed drugs available against arenaviruses; therefore, it is important to develop novel antiviral strategies to combat them, which would be facilitated by a detailed understanding of the interactions between the viruses and their hosts. To this end, we performed a transcriptomic analysis on cells infected with arenavirus lymphocytic choriomeningitis virus (LCMV), a neglected human pathogen with clinical significance, and found that the signal transducer and activator of transcription 3 (STAT3) signaling pathway was activated. A further investigation indicated that STAT3 could be activated by the RNA-dependent RNA polymerase L protein (Lp) of LCMV. Our functional analysis found that STAT3 cannot affect LCMV multiplication in A549 cells. We also found that STAT3 was activated by the Lp of Mopeia virus and Junin virus, suggesting that this activation may be conserved across certain arenaviruses. Our study explored the interactions between arenaviruses and STAT3, which may help us to better understand the molecular and cell biology of arenaviruses.

## 1. Introduction

Arenaviruses are lipid-enveloped viruses with bi-segmented, negative-stranded RNA genomes comprising a small (S) and a large (L) segment. Each RNA segment employs an ambisense coding strategy in which two proteins are encoded by two open reading frames in opposite orientations that are separated by a noncoding intergenic region (IGR) [1]. The L RNA segment encodes zinc finger matrix protein Z and the RNA-dependent RNA polymerase (RdRp) L protein (Lp), whereas the S RNA segment encodes the nucleoprotein NP and the envelope glycoprotein GPC. The GPC is cleaved into stable signal peptide (SSP), GP1, and GP2, which form spikes on the surface of the virion and mediate cell entry [2]. NP, the major structural protein, is associated with viral RNA and mediates immune evasion [3]. The Z protein can drive arenavirus budding and can inhibit viral RNA synthesis [4,5]. Lp mediates both viral genome replication and mRNA transcription [6,7].

The *Arenaviridae* family can be classified into the *Antennavirus*, *Hartmanivirus*, *Mammarenavirus*, and *Reptarenavirus* genera, while mammarenaviruses can be classified into old world (OW) and new world (NW) arenaviruses, mainly based on their antigenic properties and geographical distribution [8]. The OW arenaviruses include Lassa virus (LASV) and lymphocytic choriomeningitis virus (LCMV), while the NW arenaviruses include Junin virus (JUNV) and Machupo virus (MACV). Mammarenaviruses cause chronic and asymptomatic infections in their natural host, rodents, and several arenaviruses, such as LASV, JUNV, and MACV, cause severe hemorrhagic fever in infected humans [9]. It was reported that the population at risk of LASV infection could be as many as 200 million people [10]. The reported case fatality rate for JUNV-induced Argentine hemorrhagic fever without treatment is between 15% and 30% [11]. An increasing amount of evidence indicates that LCMV is a neglected human pathogen that is distributed worldwide [12]. LCMV usually causes asymptomatic or influenza-like symptoms in immunocompetent individuals [13]. However, the virus can be fatal to people with immune deficiency, such as patients who have undergone organ transplantation [14,15]. There are currently no FDA-licensed drugs against arenaviruses, despite their significant threat to public health. Therefore, it is important to develop novel antiviral strategies to combat arenaviruses, which would be facilitated by a detailed understanding of the interactions between arenaviruses and their hosts [16].

Signal transducer and activator of transcription (STAT) proteins are a family of transcription factors that regulate apoptosis, cell proliferation, differentiation, inflammation, and oncogenesis [17,18]. When activated by external factors, STATs are phosphorylated by receptor-associated tyrosine kinases, such as Janus kinases (JAKs), and then translocate to the nucleus to regulate the transcription of target genes [17]. STAT3 is widely studied in the field of cancer because the aberrant and persistent activation of STAT3 has been found in various types of cancers [19]. STAT3 can be activated by external factors such as IL-6 and IL-10 and helps to mediate the expression of various genes, including anti-apoptotic genes and genes involved in cell cycle progression. Recent studies have indicated that STAT3 also plays roles in a variety of chronic viral infections [20]. For example, STAT3 is essential for the life cycle of human papillomaviruses (HPVs) in keratinocytes [21]. Hepatitis C virus (HCV) activates STAT3 to upregulated genes such as *BAD* and *CCND1* to promote cellular transformation [22]. The detailed interactions between arenaviruses and STAT3 remain unclear.

In this study, we attempted to better understand the molecular and cell biology of arenaviruses by performing a transcriptomic analysis of A549 cells infected with LCMV and found that STAT3 and its target genes were upregulated. Further analysis revealed that LCMV infection promoted STAT3 phosphorylation and its transcriptional activity, and this promotion was mediated by the Lp of LCMV via the RIG-I-like receptor (RLR) signaling pathway. We also found that STAT3 can be activated by the Lp of MOPV and JUNV, suggesting that this activation may be conserved in certain arenaviruses.

## 2. Materials and Methods

### 2.1. Cells

HEK 293T, HeLa, A549, Huh-7, and BHK-21 cells were maintained in Dulbecco’s Modified Eagle’s Medium (DMEM; Thermo Fisher Scientific, Waltham, MA, USA) supplemented with 10% (*v/v*) fetal bovine serum (FBS; Gibco) in a 37 °C incubator with 5% CO_2_. BSR-T7 cells were maintained in DMEM with 10% FBS and 1 mg/mL of G418 (Sigma-Aldrich, St. Louis, MO, USA) in a 37 °C incubator with 5% CO_2_. For all of the cells used, the mycoplasma contamination was checked and excluded by a MycoBlue Mycoplasma Detector (Vazyme, Nanjing, Jiangsu, China).

### 2.2. Plasmids

The plasmid pSTAT3 used for the STAT3 reporter assay was a gift from Professor Yan-Yi Wang (Wuhan Institute of Virology, Chinese Academy of Science) [23]. Plasmid pLX304-STAT3-V5 was purchased from GE healthcare, Dharmacon. Plasmids pLX304-GFP and STAT3 Y705F and STAT3 Y705D mutants were constructed using PCR and an In-Fusion Cloning Kit (Taraka, Tokyo, Japan,639648). Plasmids for the lentivirus package and CRISPR/Cas9-based gene knockout including pMD2.G, pCMV-dR8.91, and lentiCRISPR v2 were gifts from Professor Ke Peng (Wuhan Institute of Virology, Chinese Academy of Science). Plasmids for rescuing LCMV, including pVSV-LCMV-Armstrong-S, and pVSV-LCMV-Armstrong-L, and a mini-genome system assay including pVSV-LCMV-Armstrong-S-ΔGPC/Fluc and pRL-TK (coding Renilla luciferase under CMV promoter) has been previously generated by our lab [24]. Plasmid pCAGGs-MOPV(AN20410)-Lp-strep has been generated previously [25]. Plasmids for viral protein expression, including pCAGGs-JUNV(Candid#1)-Lp-strep, pCAGGs-LCMV-GPC/NP/Z/Lp with or without C-terminal strep tag, and Lp mutants, were constructed using PCR and an In-Fusion Cloning Kit (Taraka, 639648). All of these plasmids were confirmed by Sanger sequencing.

### 2.3. Virus

LCMV (Armstrong strain, GenBank: AY847350.1 and GenBank: AY847351.1) was generated as previously reported [24]. Briefly, pVSV-LCMV-Armstrong-L (600 ng) and pVSV-LCMV-Armstrong-S (300 ng) were transfected into pre-seeded BSR-T7 cells (2 × 10^6^) to direct the intracellular synthesis of LCMV L and S antigenomes, respectively. Meanwhile, pCAGGs-LCMV-NP (300 ng) and pCAGGs-LCMV-Lp (600 ng) were co-transfected to initiate genome replication and transcription. Four days after transfection, the initially rescued viruses were transferred into BHK-21 cells for further amplification. Viral titers were determined by immunological plaque, as described in our previous study [24].

### 2.4. Reagents and Antibodies

Inhibitors MLN120B (MedChemExpress, Monmouth Junction, NJ, USA, HY-15473), Pyridone 6 (MedChemExpress, HY-14435), and Amlexanox (Selleck Chemicals, Houston, TX, USA, S3648); MagStrep XT beads (IBA Lifesciences, Göttingen, NI, Germany, 2-4090-002) and buffer BXT (IBA Lifesciences, 2-1041-250); recombinant human IL-6 protein (PeproTech, Rocky Hill, NJ, USA, 200-06-5); mouse monoclonal antibodies against GAPDH (Proteintech, Wuhan, Hubei, China, 60004-1-Ig) and V5 (Thermo Fisher Scientific, MA5-15253); rabbit monoclonal antibodies against pY705-STAT3 (Cell Signaling Technology, Danvers, MA, USA, 9145T); rabbit polyclonal antibodies against STAT3 (Proteintech, 10253-2-AP), α-Tubulin (Proteintech, 11224-1-AP), Lamin B1 (Proteintech, 12987-1-AP), and strep-tag (GenScript Biotech, Piscataway, NJ, USA, A00626-40) were purchased from the indicated companies. Rabbit polyclonal antibodies against LCMV NP were raised against NP protein (aa1-558).

### 2.5. RNA Isolation, cDNA Library Preparation, and Sequencing

A549 cells were infected with LCMV at an MOI of 0.01 or mock-treated. At 36 h post infection, cells were collected, the total RNA was extracted with Trizol (Tiangen, Beijing, China), and the RNA level was assessed with the Agilent 2100 BioAnalyzer (Agilent Technologies, Santa Clara, CA, USA) and NanoPhotometer spectrophotometer (Implen, Schatzbogen, münchen, Germany). Triplicate samples of all assays were used to construct independent libraries, followed by sequencing and analysis. The NEB Next Ultra RNA Library Prep Kit for Illumina (NEB, Ipswich, MA, USA) was used to construct the libraries for sequencing. NEB Next Poly (A) mRNA Magnetic Isolation Module kit was used to enrich the poly (A)-tailed mRNA. The mRNA was fragmented into ~200 base pair pieces. The first-strand cDNA was synthesized from the mRNA fragments using reverse transcriptase and random hexamer primers; then, the second-strand cDNA was synthesized using DNA polymerase I and RNaseH. The end of the cDNA fragment was subjected to an end repair process that included the addition of a single ‘‘A’’ base, followed by the ligation of the adapters. Products were purified and enriched by polymerase chain reaction (PCR) to amplify the library DNA. The final libraries were quantified using the Qubit 2.0 Fluorometer (Thermo Fisher Scientific) and an Agilent 2100 Bioanalyzer. After quantitative reverse transcription-PCR validation, libraries were subjected to paired-end sequencing with the pair end 150-base pair reading length on an Illumina Novaseq 6000 (Illumina, San Diego, CA, USA).

### 2.6. Data Analysis of RNA-seq

The human genome version of hg19 was used as a reference. The sequencing quality was assessed with FastQC (v0.11.8) and then low-quality data were filtered using SOAPnuke (v1.3.0). The clean reads were then aligned to the reference genome using STAR (v2.7.0d) with default parameters. The processed reads from each sample were aligned using STAR against the reference genome. The gene expression analyses were performed with Htseq (v0.11.2). These data were deposited in a GEO repository with the GEO accession number GSE173072. DESeq2 (v 1.26.0) was used to analyze the differentially expressed genes (DEGs) between the samples. The Q value was used to conduct a significance analysis. The parameters for classifying significant DEGs are 2-fold differences in the transcript abundance and *q* < 0.05.

### 2.7. Transfection and Luciferase Reporter Assays

Cells were transfected using the Lipofectamine 2000 transfection reagent (Thermo Fisher Scientific). For STAT3 overexpression, plasmid pLX304-GFP, control empty vector, pLX304-STAT3-V5, STAT3 Y705F, or STAT3 Y705D was transfected. At 48 h post transfection, cells were infected with LCMV at an MOI of 0.01 or 0.1. At 48 h post infection, cells were collected and subjected to further analysis. For the STAT3 reporter assay, pSTAT3 reporter plasmid (coding Renilla luciferase under CMV promoter and Firefly luciferase under *FOS* promoter) indicated that pCAGGs-LCMV Lp-strep mutants, pCAGGs-LCMV-Z-strep, or control empty vector were co-transfected. pCAGGs-strep (Vector) plasmids were added to ensure that each transfection received the same amount of total DNA. At 24 h post transfection, culture supernatants were replaced with DMEM without FBS. Twenty hours later, cells were lysed and measured using a dual luciferase assay kit (Promega, Madison, WI, United States) according to the manufacturer’s instructions.

### 2.8. Subcellular Fractionation

HEK 293T cells (2 × 10^6^) were collected and washed with prechilled phosphate-buffered saline (PBS). The cell pellets were resuspended in 300 μL of prechilled cytoplasm buffer (10 mM Tris-HCl, 10 mM KCl, 0.1 mM EDTA, 1 mM DTT, and 0.5 mM PMSF, with a pH of 7.5) and incubated on ice for 20 min with 15 s of vortex every 5 min. Then, 18 μL of 10% NP-40 was added. Following the vortex and another 20 min incubation on ice, the mixture was centrifugated at 4 °C, 16,000× *g* for 1 min. Then, the supernatants containing the cytoplasm fraction were harvested. The pellets were resuspended in 200 μL of prechilled nuclear extract buffer (20 mM Tris-HCl, 400 mM NaCl, 1 mM EDTA, 1 mM DTT, 1 mM PMSF, 1 mM Na_3_VO_4_, 1 mM NaF, 1 tablet protein inhibitor, pH 8.0) and rotated at 4 °C for 30 min. Followed by centrifugation at 16,000× *g* for 10 min, the supernatants containing the nuclear fraction were harvested.

### 2.9. Mini-Genome System Assay

BSR-T7 cells (3 × 10^5^) were co-transfected with pRL-TK (50 ng), pVSV-LCMV-S-ΔGPC/Fluc (300 ng), pCAGGs-LCMV-NP (300 ng), and pCAGGs-LCMV-Lp (600 ng) or mutated LCMV Lp (600 ng) plasmids by Lipofectamine 2000 transfection reagent. At 4~6 h post transfection, culture supernatants were replaced with a maintenance medium (2% FBS in DMEM). Forty-eight hours after transfection, the cells were lysed and luciferase assays were performed.

### 2.10. RNAi Experiments

For each gene, at least 2 siRNAs were designed and chemically synthesized by GenePharma (China). siRNA was transfected into HEK 293T cells using Lipofectamine RNAiMAX transfection reagent (Thermo Fisher) in a final concentration of 40 nM. Culture supernatants were replaced with maintenance medium at 24 h post transfection. Another 24 h later, cells in a well of a 24-well plate were infected with virus or re-transfected with 500 ng of indicated plasmids. siRNA without human mRNA targets was used as a negative control (NC) for RNAi-related experiments. All of the siRNA sequences are listed in Supplementary Table S1.

### 2.11. qRT-PCR

Total RNA was isolated by RNAiso Plus (Takara). RNA was reverse transcribed into cDNA using the PrimeScript RT reagent kit with gDNA Eraser (Takara). A total of 1 μL of cDNA was used for real-time PCR assay using TB Green Premix Ex Taq II (Tli RNaseH Plus) (Takara). The relative quantity of viral RNA was determined by performing a comparative Ct (ΔΔCt) experiment using *GAPDH* as an endogenous control. Gene-specific primer sequences are listed in Supplementary Table S2.

### 2.12. Co-Immunoprecipitation

For transient transfection and coimmunoprecipitation experiments, HEK 293T cells (1 × 10^7^) were transfected with 14 μg of the indicated plasmid for 36–48 h. The transfected cells were lysed in 1 mL of lysis buffer (150 mM NaCl, 50 mM Tris-HCl, 5% glycerol,1% NP40, 1 mM MgCl_2_, pH 8.0, complete protease inhibitor cocktail (Roche, Basel, BS, Switzerland)). For each immunoprecipitation, a 0.5 mL aliquot of the lysate was incubated with 10 μL of pre-cleared MagStrep XT beads at 4 °C for 2 h. Beads were washed once with 1 mL of lysis buffer and three times with 1 mL of PBS-T (0.02% Twen-20). Proteins specifically bound to beads were eluted and denatured by adding 20 μL 1 × Laemmli SDS-PAGE buffer (containing DL-Dithiothreitol) (diluted in PBS) followed by heating for 10 min at 100 °C for Western blot analysis.

### 2.13. Western Blot Analysis

For the Western blot analysis, cells were lysed in RIPA buffer (Beyotime) and denatured by adding 4× Laemmli SDS-PAGE buffer (containing DL-Dithiothreitol), followed by heating for 10 min at 100 °C. The proteins were then separated on SDS-PAGE gels and transferred onto polyvinylidene difluoride membranes by a Trans-Blot Turbo rapid transfer system (Bio-Rad, Hercules, CA, USA) according to the manufacturer’s instructions. The membranes were blocked in 5% defatted milk (dissolved in Tris-buffered saline (TBS)) for 1 h at room temperature and then incubated with a primary antibody for 1 h at room temperature or overnight at 4 °C. The membranes were then washed extensively in wash buffer (TBS containing 0.1% Tween 20) three times (for 5 min each time) with agitation and incubated with a horseradish peroxidase (HRP)-conjugated secondary antibody (Proteintech) for 1 h at room temperature according to the species source of the primary antibody. The membranes were washed three times in wash buffer and imaged using an enhanced chemiluminescence substrate solution (Sigma-Aldrich, P90720) to visualize the protein bands. GAPDH (glyceraldehyde-3-phosphate dehydrogenase) or α-Tubulin was utilized as a loading control.

### 2.14. Inhibitor Assay

HEK 293T cells (3 × 10^5^) were co-transfected with STAT3 reporter plasmid (20 ng) and pCAGGs-LCMV-Lp plasmid (480 ng). Six hours later, culture supernatants were replaced with a maintenance medium containing the indicated concentrations of MLN120B, Pyridone 6, or Amlexanox. DMSO was used as a control (vehicle). Another 42 h later, cells were lysed for a luciferase assay.

### 2.15. Cell Viability Assay

HEK 293T cells (1.5 × 10^5^) were incubated with maintenance medium containing indicated concentrations of MLN120B, Pyridone 6, or Amlexanox. DMSO was used as the control (vehicle). Forty-eight hours later, CCK-8 (Beyotime, C0039) was added and cultured for 3 h. Then, OD450 was measured to calculate the relative cell viability.

### 2.16. Establishment of CRISPR/Cas9 Based STAT3 Knock-Out A549 Cell Lines

Single-guide RNA (sgRNA) sequences targeting exons of the human *STAT3* were designed using the Millipore and Sigma CRISPR design tool (https://www.milliporesigmabioinfo.com/bioinfo_tools/faces/informatics.xhtml, accessed on 12 October 2019). A predesigned sequence with the fewest predicted off-target cleavage sites was selected (AAGACCCAGATCCAGTCCG). Oligos (forward sequence: 5′-CACCGAAGACCCAGATCCAGTCCG-3′; reverse sequence: 5′-AAACCGGACTGGATCTGGGTCTTC-3′) were synthesized, annealed, and ligated to FastDigest BsmBI (Thermo, FD0454) digested lentiCRISPR v2 plasmid using T4 DNA ligase (Thermo, EL0014). LentiCRISPR v2 plasmid containing sgRNA with no human genome target sequence (GTATTACTGATATTGGTGGG) (NC) was used as a control.

For lentivirus package, HEK 293T cells (1.5 × 10^6^) were transfected with STAT3-sgRNA LentiCRISPR v2 plasmid (800 ng) or NT-sgRNA LentiCRISPR v2 plasmid (800 ng) and two packaging plasmids, pCMV-dR8.91 (1200 ng) coding HIV-Gag and -Pol proteins and pMD2.G (400 ng) coding VSV-G protein, by a Lipofectamine 2000 transfection reagent. The culture medium was replaced with a maintenance medium 12 h after transfection. After an additional 36 h, the supernatant containing recombinant virus was filtered by a 0.22 μm PES filter and used to infect A549 cells three subsequent times in the presence of polybrene (8 μg/mL) for a higher transduction efficiency. Four days after the first transduction, cells were selected by incubating with 1 μg/mL of puromycin for another 10 days, then a single cell was isolated by serial dilutions and allowed to expand for 2 to 3 weeks without puromycin. Genomic DNA from the cell lines was extracted using a TIANamp genomic DNA kit (Tiangen, DP304). The modified regions were amplified using specific genomic cleavage detection primers (forward sequence: 5′-ACTTTCCGAATGCCTCCTCC-3′; reverse sequence: 5′-TGCTTTGTTTTGCTTGCTCC-3′). PCR production was ligated to T-vector using 5 × TA/Blunt-Zero Cloning Kit (Vazyme, C601-01), followed by transforming into NEB stable bacteria. At least 10 bacteria clones of each transformation were defined by Sanger sequencing and A549 cell lines exhibiting frameshift mutations at the corresponding sites were selected and further confirmed by Western blotting. Three isogenic cell lines bearing the desired *STAT3* gene editing outcomes and one control cell line (NC) were selected for further study.

## 3. Results

### 3.1. LCMV Infection Activates the STAT3 Signaling Pathway

**Figure 1:**
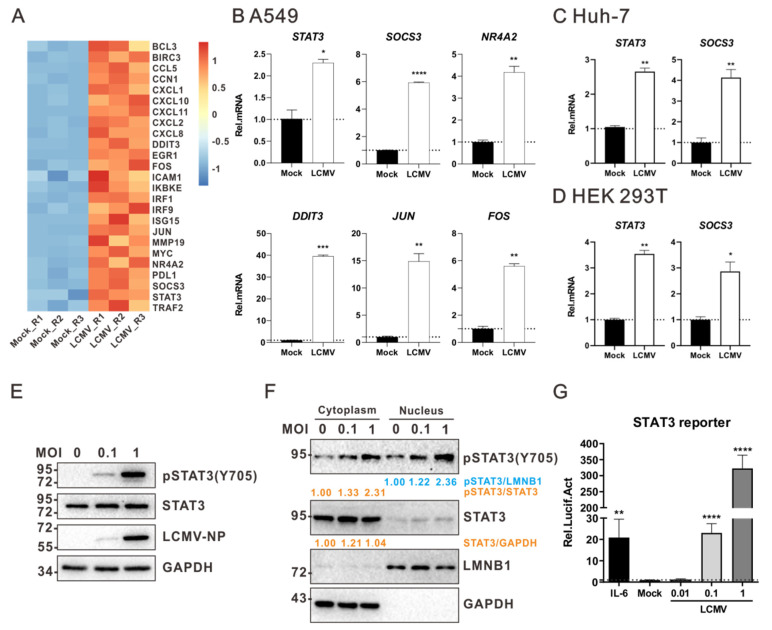
LCMV infection activates STAT3 signaling pathway. (**A**) Transcriptomic analysis of LCMV-infected A549 cells. A549 cells were infected with LCMV at an MOI of 0.01 or mock-treated. Thirty-six hours later, the cells were collected. Total mRNA was extracted and subjected to RNA-seq sequencing. (**B**–**D**) LCMV infection induces the transcription of *STAT3* and its target genes on A549 (**B**), Huh-7 (**C**), or HEK 293T (**D**) cells. A549 and Huh-7 cells were infected with LCMV at an MOI of 0.01. HEK 293T cells were infected with LCMV at an MOI of 1. At 24 h p.i., cells were collected for total RNA extraction and qRT-PCR experiments. *GAPDH* mRNA levels were used as an endogenous control. (**E**) LCMV infection activates the phosphorylation of STAT3 Y705. HEK 293T cells were infected with LCMV at the indicated MOIs. At 24 h p.i., cells were lysed for Western blot analysis. (**F**) LCMV infection promotes the nuclear translocation of pSTAT3 (Y705). HEK 293T cells were infected with LCMV. At 24 h p.i., cells were collected and then fractionated into nuclear and cytoplasmic fractions for Western blot analysis. To normalize the STAT3 protein intensity, lamin B1 (LMNB1) and GAPDH were used as the nuclear and cytoplasmic endogenous controls, respectively. Relative protein intensity of pSTAT3 and STAT3 was analyzed using the ImageJ software. (**G**) LCMV infection activates STAT3 reporter activity. HEK 293T cells were transfected with the pSTAT3 reporter plasmid, and at 24 h post transfection, cells were infected with LCMV at the indicated MOIs. At 12 h post infection, cell was lysed and subjected to luciferase assays to measure the STAT3 reporter activity. IL-6 treatment was used as a positive control. A representative from at least three independent experiments is shown. Graphs show mean ± SD. (*n* = 3, **B**–**D**,**G**). * *p* < 0.05, ** *p* < 0.01, *** *p* < 0.001, **** *p* < 0.0001, unpaired Student’s *t*-test.

To explore how LCMV infection regulates host gene expression, a transcriptomic analysis of LCMV-infected cells was performed. Briefly, A549 cells permissive for LCMV were infected with LCMV at a multiplicity of infection (MOI) of 0.01. Cells were collected at 36 h post infection (p.i.), and the total RNA was extracted and subjected to transcriptomic analysis. We found that multiple host genes were differentially regulated, including genes involved in the establishment of protein localization to organelle, purine ribonucleotide metabolic process, response to virus (such as IFN-I and ISGs), etc. (Figure S1). Among these regulated genes, the expressions of STAT3 and multiple STAT3 target genes were upregulated, including *SOCS3*, *NR4A2,* and *MMP19* (Figure 1A). Then, using quantitative real-time PCR (qRT-PCR), we found that the intracellular mRNA levels of *SOCS3, NR4A2, DDIT3, JUN,* and *FOS* were upregulated as a result of the LCMV infection of A549 cells (Figure 1B), suggesting that LCMV may activate the STAT3 signaling pathway. We also found that intracellular mRNA levels of *STAT3* and *SOCS3* were upregulated in LCMV-infected Huh-7 (Figure 1C) and HEK 293T (Figure 1D), another two LCMV-permissive cell lines.

A hallmark of the activation of the STAT3 signaling pathway is the phosphorylation of STAT3 Y705 (pSTAT3 Y705) [26], which, thereafter, promotes the translocation of the STAT3 protein from the cytoplasm to the nucleus to activate the transcription of target genes [27]. Here, using Western blotting, we found that LCMV infection promoted the phosphorylation of STAT3 Y705 (Figure 1E) and nuclear translocation in HEK 293T cells (Figure 1F). To confirm the activation of STAT3 signaling in response to LCMV infection, a reporter system was employed in which the expression of the firefly luciferase gene was controlled by a high-affinity version of the serum-inducible element (hSIE) from the *c-fos* promoter, which has previously been confirmed to be STAT3-activated [23,28]. We observed the expression of the reporter in response to the treatment of IL-6, providing evidence for the activation of the STAT3 and validating the STAT3 reporter system used here. We also found LCMV infection can activate the STAT3 reporter (Figure 1G), suggesting that LCMV infection can activate the STAT3 signaling pathway.

### 3.2. The Activation of the STAT3 Signaling Pathway Is Mediated by the L Protein of LCMV

**Figure 2:**
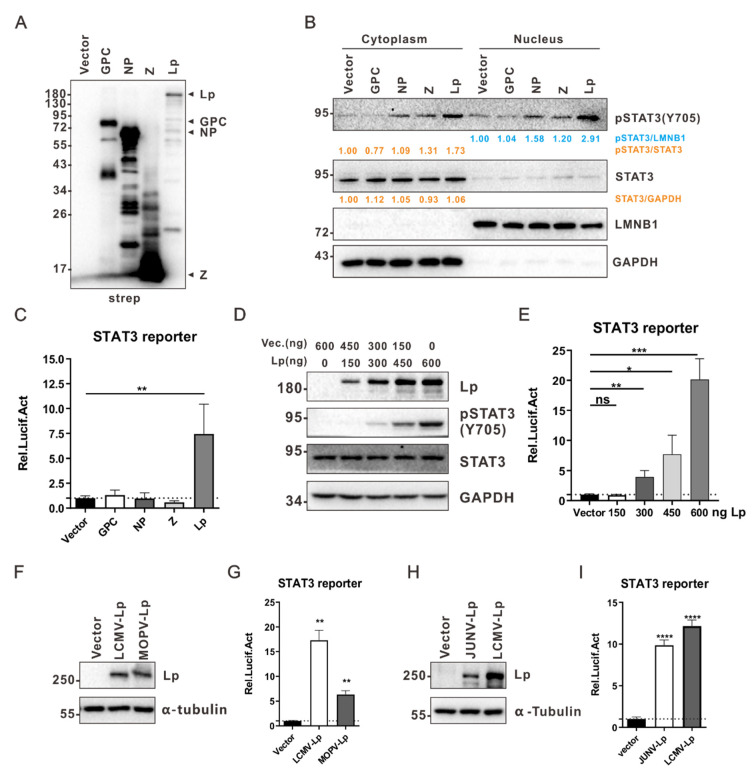
The activation of STAT3 signaling pathway is mediated by the L protein of LCMV. (**A**) HEK 293T cells in a well of a 6-well plate were transfected with 2.5 μg of the indicated step-tag pCAGGs plasmids. At 36 h post transfection, cells were lysed and proteins were subjected to Western blot analysis. Expression levels of all viral proteins were detected with a mouse monoclonal antibody against the strep tag. (**B**) LCMV Lp promotes the nuclear translocation of pSTAT3 (Y705). HEK 293T cells were transfected with plasmids as in (**A**). At 36 h post transfection, cells were collected and then fractionated into nuclear and cytoplasmic fractions for Western blot analysis. To normalize the STAT3 protein intensity, lamin B1 and GAPDH were used as the nuclear and cytoplasmic endogenous loading controls, respectively. Relative protein intensity was analyzed using the ImageJ software. (**C**) LCMV Lp activates STAT3 reporter activity. HEK 293T cells in a well of a 24-well plate were transfected with STAT3 reporter plasmid (20 ng) and indicated viral protein-expressing plasmids (480 ng). At 36 h post transfection, cells were lysed and subjected to luciferase assays to measure the STAT3 reporter activity. (**D**,**E**) LCMV Lp activates STAT3 in a dose-dependent manner. HEK 293T cells were transfected with STAT3 reporter plasmid (**E**) and an indicated dose of pCAGGs-LCMV-Lp-strep plasmids. pCAGGs-strep (Vector) was added to ensure that each transfection received the same amount of total DNA. At 36 h post transfection, cells were lysed and subjected to luciferase assays to measure the STAT3 reporter activity. (**F**–**I**) The effect of MOPV-Lp or JUNV-Lp on STAT3 activation. HEK 293T cells in a well of a 24-well plate were transfected with 20 ng of STAT3 reporter plasmid (**G**,**I**) and 480 ng of pCAGGs-strep (Vector), pCAGGs-LCMV-Lp-strep, pCAGGs-MOPV-Lp-strep, or pCAGGs-JUNV-Lp-strep. The expression of Lp was measured by Western blotting (**F**,**H**), while the STAT3 reporter activity was measured by a luciferase assay (**G**,**I**). A representative from at least two independent experiments is shown. Graphs show mean ± SD. (*n* = 3, **C**,**E**,**G**,**I**) * *p* < 0.05, ** *p* < 0.01, *** *p* < 0.001, **** *p* < 0.0001, ns not significant, unpaired Student’s *t*-test.

Previous studies have indicated that the STAT3 signaling pathway can be manipulated by viral proteins, such as rabies virus P protein [29], human papillomavirus E6 protein [30], and hepatitis E virus ORF3 protein [31]. Here, to explore which LCMV proteins can affect the STAT3 signaling pathway, HEK 293T cells were transfected with plasmids expressing GPC, NP, Lp, or Z (Figure 2A); then, 36 h later, the phosphorylation of STAT3 Y705 was examined. As shown in Figure 2B, the expression of Lp can induce the phosphorylation of STAT3 Y705 significantly, while GPC, NP, or Z did not have the same effect. We then detected STAT3 reporter activity and found that Lp, but not other viral proteins, can activate the STAT3 reporter (Figure 2C), suggesting that the activation of the STAT3 signaling pathway is mediated by the Lp of LCMV. We also found that LCMV Lp activated the STAT3 signaling pathway in a dose-dependent manner (Figure 2D,E). We further examined whether the Lp of other arenaviruses can activate the STAT3 signaling pathway. To this end, HEK 293T cells were transfected with plasmids expressing an Lp of MOPV or JUNV (Figure 2F,H), and STAT3 reporter activity was measured at 24 h post transfection. As shown in Figure 2G,I, MOPV Lp and JUNV Lp can activate STAT3 reporter activity, suggesting that the STAT3 signaling pathway can be activated by the Lp of certain arenaviruses.

**Figure 3:**
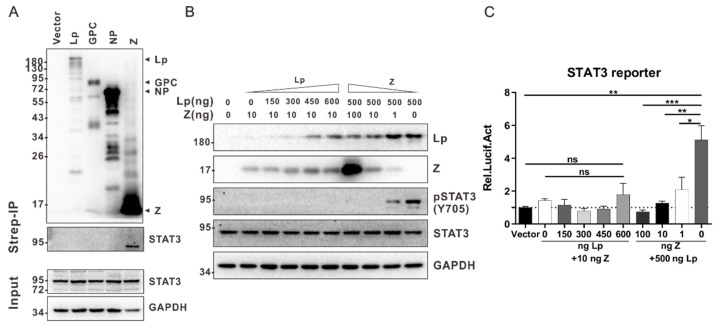
The Lp-mediated activation of STAT3 signaling pathway can be attenuated by Z. (**A**) LCMV Z interacts with STAT3. HEK 293T cells in a 10 cm cell culture dish were transfected with 14 μg of the indicated pCAGGs-strep plasmids. At 36 h post transfection, cells were lysed and subjected to a co-immunoprecipitation assay. The precipitates were analyzed by Western blot analysis. (**B**,**C**) LCMV Z inhibits the activation of STAT3 by decreasing the expression of LCMV Lp. HEK 293T cells were transfected with an indicated dose of pCAGGs-LCMV-Z-strep, pCAGGs-LCMV-Lp-strep, STAT3 reporter plasmids (**C**), and pCAGGs-strep (Vector) plasmids. pCAGGs-strep (Vector) plasmids were added to ensure that each transfection received the same amount of total DNA. At 36 h post transfection, the cells were lysed and analyzed by Western blotting (**B**) or luciferase assays (**C**). A representative from at least two independent experiments is shown. Graphs show mean ± SD. (*n* = 3, **C**). * *p* < 0.05, ** *p* < 0.01, *** *p* < 0.001, ns not significant, unpaired Student’s *t*-test.

It has been reported that viral proteins can affect the STAT3 signaling pathway by interacting with STAT3 [21,29,30,31,32,33,34,35,36,37,38]. Hence, we explored whether LCMV proteins can interact with STAT3. HEK 293T cells were transfected with plasmids expressing viral proteins, and possible interactions between viral proteins and STAT3 were detected by coimmunoprecipitation and Western blotting. As shown in Figure 3A, the Z protein can interact with STAT3, while no interaction was observed between STAT3 and other viral proteins. However, considering that the expression level of Lp is low here, we cannot exclude the possibility that the interaction may exist when Lp is expressed at a high level. We further found that the expression of Z can attenuate the Lp-mediated activation of the STAT3 signaling pathway in a dose-dependent manner (Figure 3B,C). It has been reported that arenavirus Z can reduce intracellular Lp levels [39], and indeed, we found that the intracellular level of Lp decreased as a result of the Z expression here (Figure 3B), suggesting that the attenuation of the Lp-mediated activation of the STAT3 signaling pathway observed here might be partially attributed to the Z-mediated downregulation of the intracellular Lp levels.

### 3.3. Activation of the STAT3 Signaling Pathway by Lp Is Associated with Its RdRp Activity

**Figure 4:**
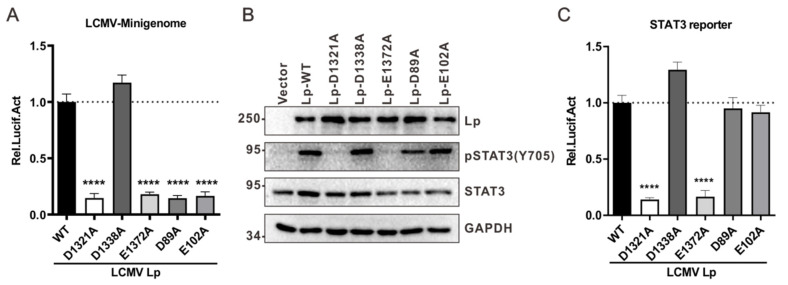
The activation of the STAT3 signaling pathway by Lp is associated with its RdRp activity. (**A**) Site mutations on LCMV Lp affect its mini-genome activity. BSR-T7 cells in a well of a 24-well plate were co-transfected with 50 ng pRL-TK, 300 ng pVSV-LCMV-S-ΔGPC/Fluc, 300 ng pCAGGs-LCMV-NP, and 600 ng of the indicated pCAGGs-LCMV-Lp plasmids. At 48 h post transfection, cells were lysed and subjected to luciferase assay to measure their mini-genome activity. (**B**,**C**) The activation of STAT3 is associated with the RNA-dependent RNA polymerase activity, but not the endonuclease activity of LCMV Lp. (**B**) HEK 293T cells in a well of a 24-well plate were transfected with 500 ng of the indicated pCAGGs-LCMV-Lp plasmids. At 36 h post transfection, cells were lysed and subjected to Western blot analysis. (**C**) HEK 293T cells were transfected with the indicated pCAGGs-LCMV-Lp and STAT3 reporter plasmids. At 36 h post transfection, cells were lysed and subjected to a luciferase assay to measure the STAT3 reporter activity. A representative from at least three independent experiments is shown. Graphs show mean ± SD. (*n* = 3, **A**,**C**). **** *p* < 0.0001, unpaired Student’s *t*-test.

Lp is a multidomain protein with both transcription and replication activities [6]. The transcription process requires a host mRNA-derived primer to be captured by Lp through cap snatching [40], which is mediated by the amino (N)-terminal endonuclease domain of the Lp. To determine whether the activation of the STAT3 signaling pathway by Lp is associated with its RdRp activity or cap snatching activity, we induced loss-of-function mutations in the endonuclease active site (D89 and E102) or RdRp active sites (D1321 and D1372) and examined their effect on the STAT3 signaling pathway [25,41]. D1338, which was near the RdRp active site but did not affect the RdRp activity (Figure 4A), was chosen as a negative control. As shown in Figure 4, mutations resulting in a loss of either RdRp or endonuclease activity led to the inhibition of LCMV mini-genome (MG) activity, while mutations in the RdRp active sites (D1321A and D1372A) but not in the endonuclease active site (D89A and E102A), resulted in the reduced activation of the STAT3 signaling pathway, suggesting that the activation of the STAT3 signaling pathway by Lp is associated with its RdRp activity.

### 3.4. RIG-I Signaling Pathway Is Important for the Lp-Mediated Activation of STAT3

**Figure 5:**
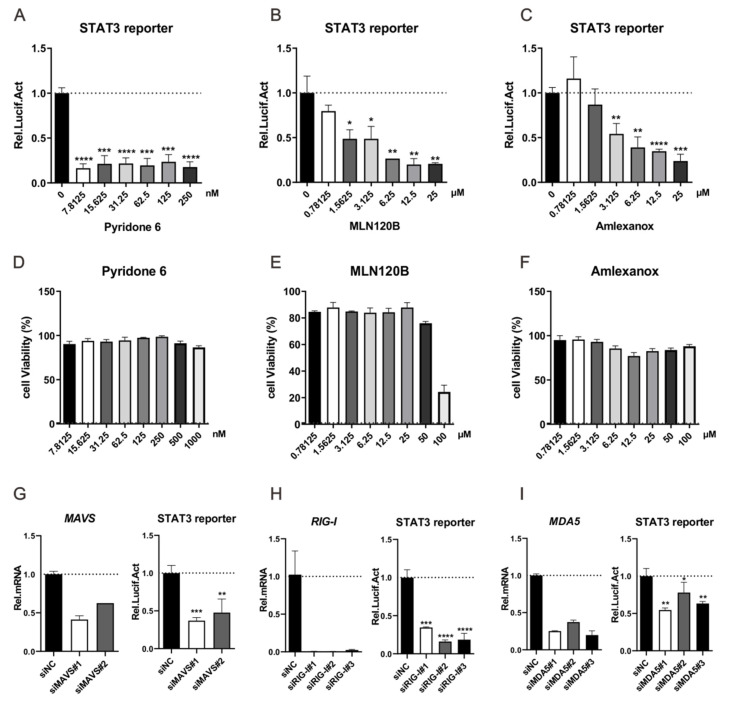
The activation of the STAT3 signaling pathway by Lp is mediated by the RIG-I-like signaling pathway. (**A**) Pan-JAK inhibitor Pyridone 6 inhibits the activation of STAT3 induced by LCMV Lp. HEK-293T cells in a well of a 24-well plate were co-transfected with 480 ng of pCAGGs-LCMV-Lp and 20 ng STAT3 reporter plasmids. At 6 h post transfection, cells were treated with the indicated concentration of Pyridone 6. At 36 h post transfection, the cells were lysed to measure the STAT3 reporter activity using a luciferase assay. (**B**) IKK-β inhibitor MLN120B inhibits the activation of STAT3 induced by LCMV Lp. HEK-293T cells in a well of a 24-well plate were co-transfected with 480 ng of pCAGGs-LCMV-Lp and 20 ng of STAT3 reporter plasmids. At 6 h post transfection, cells were treated with the indicated concentration of MLN120B. At 36 h post transfection, the cells were lysed to measure the STAT3 reporter activity using a luciferase assay. (**C**) TBK1 and IKK-ε inhibitor Amlexanox inhibits the activation of STAT3 induced by LCMV Lp. HEK-293T cells in a well of a 24-well plate were co-transfected with 480 ng of pCAGGs-LCMV-Lp and 20 ng of STAT3 reporter plasmids. At 6 h post transfection, cells were treated with the indicated concentration of Amlexanox. At 36 h post transfection, the cells were lysed to measure the STAT3 reporter activity using a luciferase assay. (**D**–**F**) The effect of Pyridone 6, MLN120B, and Amlexanox on the viability of HEK-293T cells. (**G**–**I**) The effect of the knockdown of RIG-I, MDA5, or MAVS on the activation of STAT3 mediated by LCMV Lp. HEK-293T cells in a well of a 24-well plate were trans-transfected with the indicated siRNAs. At 24 h post transfection, cells were transfected with 20 ng of STAT3 reporter plasmid and 480 ng of pCAGGs-LCMV-Lp. Thirty-six hours later, the cells were collected. Intracellular RNA was extracted and the intracellular level of RIG-I, MDA5, or MAVS was measured using qRT-PCR (left). For luciferase assay, cells were collected and the intracellular level of STAT3 reporter activity was measured (right). A representative from at least three independent experiments is shown. Graphs show mean ± SD. (*n* = 3, **A**–**I**). * *p* < 0.05, ** *p* < 0.01, *** *p* < 0.001, **** *p* < 0.0001, unpaired Student’s *t*-test.

STAT3 can be activated by a wide range of ligands that bind to cytokines or growth factor receptors [20]. Upon cytokine binding, there is typically recruitment and a reciprocal transphosphorylation of tyrosine kinases of the JAK family comprising JAK1, JAK2, JAK3, and tyrosine kinase 2 (TYK2) [42], and these kinases, in turn, recruit and phosphorylate STAT3 at the highly conserved tyrosine residue Y705 [26]. We then tested whether the activation of STAT3 by Lp is mediated by JAKs. To this end, Lp-expressing cells were treated with pyridone 6, a pan-JAKs inhibitor [43]. As shown in Figure 5A,D, Lp expression activates STAT3 reporter activity, while pyridone 6 treatment can repress this activation, suggesting that the activation of the STAT3 by Lp is mediated by JAKs (Figure S2).

It has been reported that several cytokines encoded by NF-κB target genes, most notably interleukin-6 (IL-6), are important STAT3 activators [44]. We explored whether the activation of STAT3 is mediated by NF-κB. Briefly, Lp-expressing HEK 293T cells were treated with MLN120B, an inhibitor of NF-κB [45], and STAT3 reporter activity was measured after 36 h. As shown in Figure 5B,E, the treatment of MLN120B can repress Lp-activated STAT3 reporter activity, suggesting that the activation of STAT3 by Lp can be mediated by NF-κB (Figure S2).

Our previous study indicated that arenavirus Lp can activate the production of type I interferon (IFN-I) [25], which can reportedly activate STAT3 phosphorylation. IFN-I production is mainly mediated by the RIG-I signaling pathway in HEK 293T cells. Here, we found that amlexanox, an inhibitor of IKKε [46], which is essential for IFN-I production in the RLR signaling pathway, reduced the Lp-mediated activation of STAT3 (Figure 5C,F).

During viral infection, NF-κB and IKKε can be activated by the Toll-like receptor (TLR) or RLR signaling pathway. Considering that the TLR expression level is low in HEK 293T cells [47], the activation of NF-κB and IKKε may mainly be mediated by RLR. As an important adaptor protein, MAVS is located upstream of NF-κB and IKKε in the RLR signaling pathway. We examined the effects of MAVS knockdown on Lp-mediated STAT3 activation. The transfection of two siRNAs designed against MAVS demonstrated their ability to decrease the intracellular mRNA levels of *MAVS* and they were therefore used in subsequent analyses. HEK 293T cells were co-transfected with plasmids expressing Lp and siRNAs against MAVS, and STAT3 promoter activity was detected after 36 h. As shown in Figure 5G, MAVS knockdown blocked the Lp-mediated activation of the STAT3 reporter, suggesting that MAVS is important for the Lp-mediated activation of STAT3 (Figure S2).

Next, we examined whether RIG-I and MDA5, two proteins upstream of MAVS in the RLR signaling pathway, affect Lp-mediated STAT3 activation (Figure 5H,I). First, siRNAs were designed against RIG-I and MDA5 and their knockdown efficiencies were tested. HEK 293T cells were then co-transfected with plasmids expressing Lp and siRNAs against RIG-I or MDA5. The activation of STAT3 was assessed by measuring the STAT3 reporter activity. As shown in Figure 5H,I, the knockdown of RIG-I or MDA5 inhibited Lp-mediated STAT3 activation, suggesting that RIG-I and MDA5 are important for Lp-mediated STAT3 activation (Figure S2).

### 3.5. Impairment of STAT3 Signaling Pathway Does Not Affect LCMV Multiplication in A549 Cells

**Figure 6:**
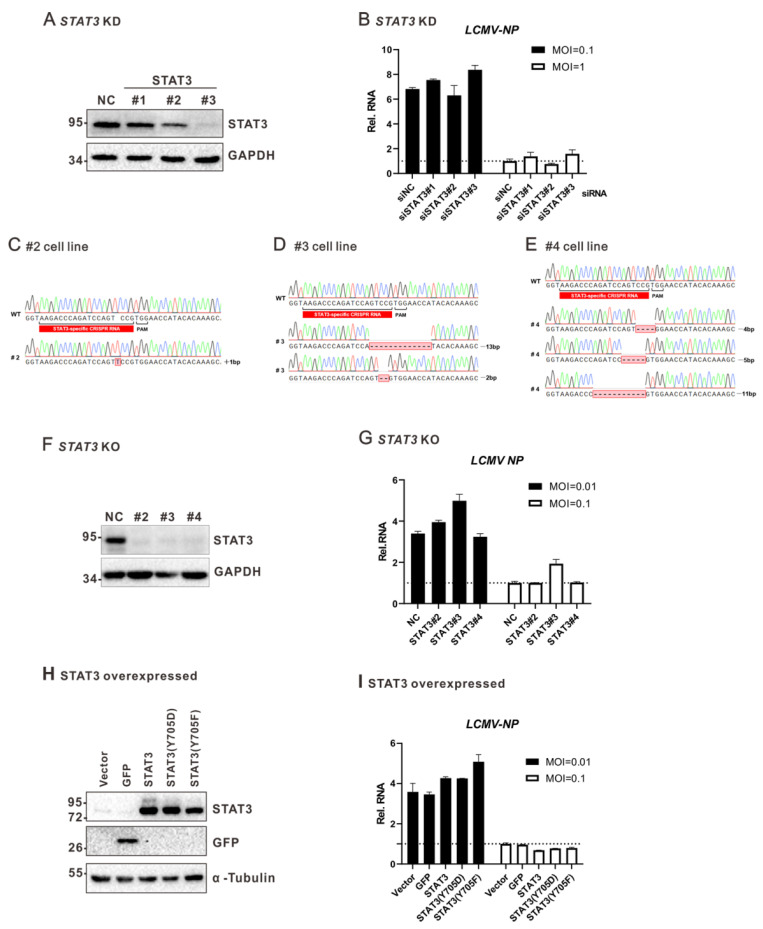
STAT3 does not affect LCMV multiplication in A549 cells. (**A**) RNAi-based knockdown of STAT3 in A549 cells. (**B**) The effect of the knockdown of STAT3 on LCMV multiplication. STAT3-knockdown A549 cells were infected with LCMV at an MOI of 0.1 or 1. At 24 h post infection, cells were collected and the intracellular level of LCMV RNA was measured with qRT-PCR using primers targeting the NP ORF sequence. Intracellular levels of *GAPDH* mRNA were used as the endogenous control. (**C**–**E**) Sanger sequencing analysis of three CRISPR/Cas9-based STAT3-knockout cell lines. (**F**) Immunoblotting analysis of intracellular STAT3 protein level in STAT3-knockout A549 cells. (**G**) The effect of the knockout of STAT3 on LCMV multiplication. STAT3-knockout A549 cells were infected with LCMV at an MOI of 0.01 or 0.1. At 48 h post infection, cells were collected and the intracellular level of LCMV RNA was measured with qRT-PCR. Intracellular levels of *GAPDH* mRNA were used as the endogenous control. (**H**) Immunoblotting analysis of STAT3 protein level in cells overexpressing STAT3, STAT3 Y705D, or STAT3 Y705F. (**I**) The effect of the overexpression of STAT3, STAT3 Y705D, or STAT3 Y705F on LCMV multiplication. Cells overexpressing STAT3, STAT3 Y705F, or STAT3 Y705D were infected with LCMV at an MOI of 0.01 or 0.1. At 48 h post infection, cells were collected and the intracellular level of LCMV RNA was measured with qRT-PCR. Intracellular levels of GAPDH mRNA were used as the endogenous control. A representative from at least two independent experiments is shown. Graphs show mean ± SD (*n* = 3, **B**,**G**,**I**).

Recent studies have shown that STAT3 plays important roles in viral replication, including HCV, varicella zoster virus (VZV), and human cytomegalovirus (HCMV) [18,48,49,50]. To explore whether STAT3 can affect LCMV multiplication, A549 cells were transfected with siRNA against STAT3 and infected with LCMV. Forty-eight hours post infection, cells were collected. As shown in Figure 6A,B, the knockdown of STAT3 did not affect the intracellular level of LCMV RNA. We further generated STAT3 knockout cells by using the CRISPR-Cas9 system to explore the roles of STAT3 in LCMV multiplication. Briefly, lentiviruses coding Cas9 and sgRNAs targeting the STAT3 gene were transduced into A549 cells to direct genome editing. The knockout of STAT3 was confirmed by sequencing (Figure 6C–E, frameshift mutations at the corresponding sites) and Western blot analysis (Figure 6F). As shown in Figure 6G, the knockout of STAT3 did not affect the LCMV intracellular level of LCMV RNA. We also examined whether the overexpression of STAT3 could affect LCMV multiplication. STAT3 Y705F is a dominant-negative mutant of STAT3 [23], while STAT3 Y705D is a phospho-mimetic form of STAT3 [51]. Cells were transfected with plasmids encoding STAT3, STAT3 Y705F, or STAT3 Y705D, and the overexpression of STAT3 was confirmed by Western blotting (Figure 6H). As shown in Figure 6I, the overexpression of STAT3, STAT3 Y705D, or STAT3 Y705F did not affect the intracellular level of LCMV RNA, suggesting that the STAT3 signaling pathway is not essential for LCMV multiplication in A549 cells.

## 4. Discussion

STAT3 is a transcription factor that can be activated by cytokines, growth factor receptors, and nonreceptor-like tyrosine kinases [18]. Following activation, STAT3 translocates into the nucleus to regulate the expression of target genes involved in cell proliferation, differentiation, apoptosis, and oncogenesis. Recent studies have shown that STAT3 plays important roles in viral infection and pathogenesis [18]. One study indicated that STAT3 is actively phosphorylated by HCV, and the expression of a constitutively active form of STAT3 promotes HCV replication, while the chemical inhibition or knockdown of STAT3 represses HCV replication [48]. VZV triggers STAT3 phosphorylation in cells infected in vitro as well as in human skin xenografts in mice in vivo, and chemical inhibitors of STAT3 restrict VZV replication in vitro [49]. The interactions between arenavirus and the STAT3 signaling pathway have not been characterized.

Viral RdRp proteins, which are the largest proteins encoded by viral genomes in most RNA viruses, mainly participate in viral genome replication and mRNA transcription. In recent years, viral RdRp proteins have also been reported to participate in multiple processes other than RNA synthesis. For example, the RdRp protein of tick-borne encephalitis virus (TBEV) induces the expression of RANTES, which can induce leukocytes, human blood monocytes, and T lymphocyte migration [52]. The RdRp protein of Zika virus, NS5, can inhibit the IFN-I signaling pathway by interacting with STAT2 [53,54], while the RdRps of HCV and Semliki Forest virus (SFV) produce dsRNAs in transfected cells to activate the RLR signaling pathway and induce IFN-I production [55,56], suggesting the multiple potential functions of viral RdRp.

The RdRps of arenaviruses usually comprise more than 1000 amino acids and can be divided into three domains, similar to the influenza virus polymerase tri-subunits: the N-terminal PA-like region, the central RdRp (PB1-like) region, and the C-terminal PB2-like region [6]. During virus infection, viral mRNA synthesis is primed using short methyl-7-guanosine (m7G)-capped RNA sequences, which the viral polymerase cleaves from host RNA polymerase II transcripts in a process known as ‘‘cap snatching’’ [57], and this process is mediated by the N- and C-terminal domains of arenaviruses [40,58,59,60]. Recent studies have indicated the functions of arenavirus Lp other than in viral RNA replication and transcription. For example, our previous study indicated that the Lp of the arenavirus Mopeia virus (MOPV) can activate the production of IFN-I [25]. A recent study indicated that the Lp of LASV may produce RNAs with ORFs containing a combination of both host and viral sequences, and these chimeric host–virus transcripts may produce novel host–virus-encoded proteins that activate T cell responses [57]. Here, we found that LCMV can activate the STAT3 signaling pathway, and this process is mediated by Lp. Further study indicated that the RdRp activity of Lp accounted for the activation of the STAT3 signaling pathway. RdRp activity may activate both RIG-I and MDA5, thereafter activating IRF3/7 and p50/p65 to induce the production of IFN-I and NF-κB to induce the production of cytokines. Secreted IFN-I and cytokines then bind their receptors to activate the tyrosine kinases of the JAK family and subsequently the STAT3 signaling pathway (Figure S2). However, the detailed mechanism through which the RdRp activity of Lp activates RIG-I and MDA5 requires further study and exploration. We found that Z can repress Lp-mediated STAT3 activation, which might be partially mediated by decreasing the intracellular level of Lp (Figure 3). However, these phenomena need to be confirmed in the context of LCMV infection.

We found that the Lp of MOPV or JUNV can also activate the STAT3 signaling pathway, while LASV Lp can activate the STAT3 reporter but cannot induce the phosphorylation of STAT3 Y705 (data not shown), suggesting that this phenomenon may be conserved among certain arenaviruses. Although it has been reported that STAT3 can affect the replication of numerous viruses, including HCV [48], we found that the knockdown of STAT3 by RNAi or knockout of STAT3 by CRISPR or the overexpression of STAT3 did not affect the LCMV multiplication in A549 cells (Figure 6), suggesting that the activation of the STAT3 signaling pathway by LCMV may not promote or inhibit its replication. Whether STAT3 can affect LCMV in other cell lines or in vivo needs to be studied further. STAT3 regulates a wide spectrum of biological processes, including inflammation, tissue regeneration, cell proliferation, cell survival, cellular differentiation, angiogenesis, chemotaxis, and cell adhesion [20]. For example, a recent study indicated that STAT3 is phosphorylated as a result of infection with Rift Valley fever virus (RVFV). A loss of STAT3 did not affect viral replication but did result in cells being more susceptible to RVFV-induced cell death [61]. The biological consequences of STAT3 activation by LCMV might have potentially serious outcomes for host organisms.

## Data Availability

The transcriptomic data in this study were submitted to GEO repository (accession number GSE173072).

## Supplementary Materials

The following are available online at https://www.mdpi.com/article/10.3390/v13060976/s1, Figure S1: Gene ontology analysis of differentially regulated genes in LCMV infected A549 cells based on biological process. Figure S2: A proposed model for the activation of STAT3 by the LCMV Lp. Table S1: siRNAs used in RNAi experiments. Table S2: Primers used in qPCR experiments.
