# Supplementary material for: Activation of the STAT3 Signaling Pathway by the RNA-Dependent RNA Polymerase Protein of Arenavirus
Source: Viruses. 2021 May 25;13(6):976. doi: 10.3390/v13060976 (PMC8225222; doi:10.3390/v13060976)
Supplement: Supplementary file 1 [file viruses-13-00976-s001.zip › viruses-1216439-supplementary.pdf]

Supplementary Information

Supplementary Tables.

Supplementary Table S1. siRNAs used in RNAi experiments.

Name	Forward sequence (5'-3')
siNC	UUCUCCGAACGUGUCACGUTT
siMAVS#1	AGAGGAGAAUGAGUAUAAGTT
siMAVS#2	UACCAAGGGUUGGAUAUAUTT
siRIG-I#1	AAGCCUUCCAGGAUUAUAUTT
siRIG-I#2	CACGGAUUAGCGACAAAUUTT
siRIG-I#3	CACUUGUGGACGCUUUAAATT
siMDA5#1	CAAGGAGUUCCAACCAUUUTT
siMDA5#2	AACAAAGAAGCAGUGUAUATT
siMDA5#3	AUCGUUUGAGAACGCUCAUTT
siSTAT3#1	AAAGAAUCACAUGCCACUUTT
siSTAT3#2	ACAAUCUACGAAGAAUCAATT
siSTAT3#3	CGUCCAGUUCACUACUAAATT

Supplementary Table S2. Primers used in qPCR experiments.

Gene	Forward sequence (5'-3')	Reverse sequence (5'-3')
GAPDH	GAAGGTGAAGGTCGGAGTC	GAAGATGGTGATGGGATTTC
SOCS3	ACCTACTGAACCCTCCTCCG	TGACGGTCTTCCGACAGAGA
STAT3	CAGCGGTAAGACCCAGATCC	TGGTATTGCTGCAGGTCGTT
LCMV-NP	GTACAAGCGCTCACAGACCT	GTTACCCCCATCCAACAGGG
MAVS	GCCGTTTGCTGAAGACAAGA	TTCCCTGAGAGTGTGCAGGT
MDA5	CTGTGCTGGACTACCTGACC	GAGTGGGCTGAAGGAGGTTC
RIG-I	TCCTTTATGAGTATGTGGGCA	TCGGGCACAGAATATCTTTG
JUN	TGCCTCCAAGTGCCGAAAAA	CTGCTGCGTTAGCATGAGTT
FOS	GCCTCTCTTACTACCACTCACC	AGATGGCAGTGACCGTGGGAAT
DDIT3	TTCACCACTCTTGACCCTGC	TTCCTGCTTGAGCCGTTCAT
NR4A2	AGTATGGGTCCTCGCCTCAA	TGGCTGTGTTGCTGGTAGTT

Supplementary Figures.

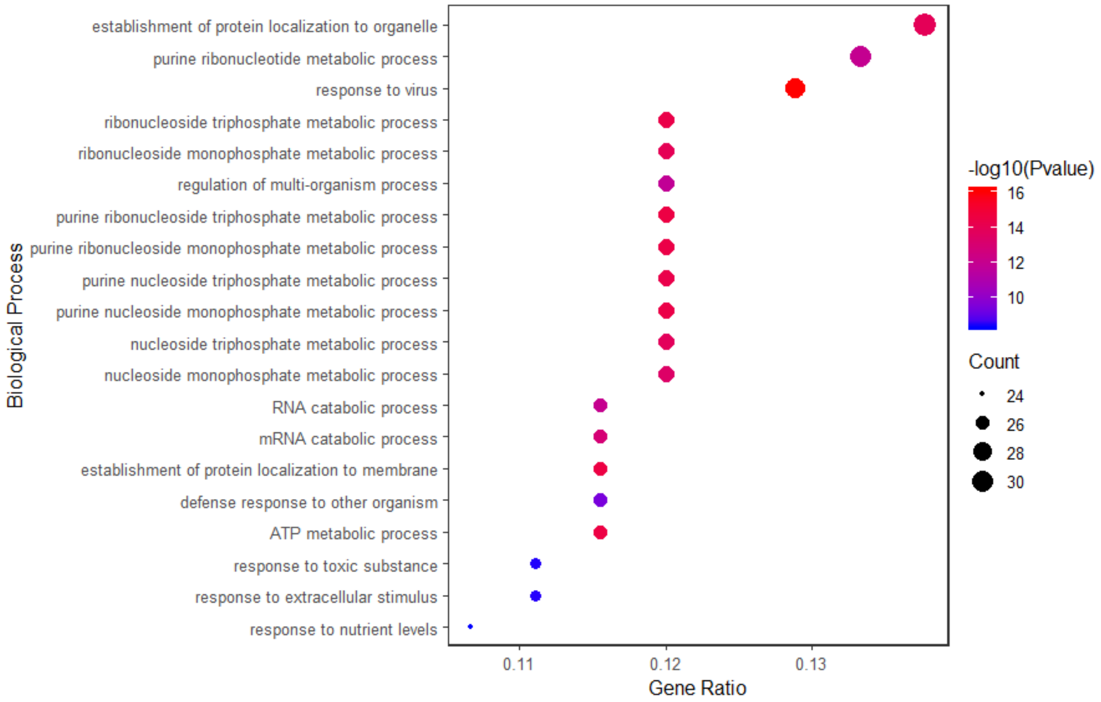

Figure S1. Gene ontology analysis of differentially regulated genes in LCMV infected A549 cells based on biological process.

A549 cells were infected with LCMV at an MOI of 0.01 or mock-treated. Thirty-six hours later, intracellular mRNA was extracted for transcriptomic sequencing. Different regulated genes (change fold > 2, q < 0.05) were subjected to gene ontology analysis. TOP 20 enriched biological processes were performed.

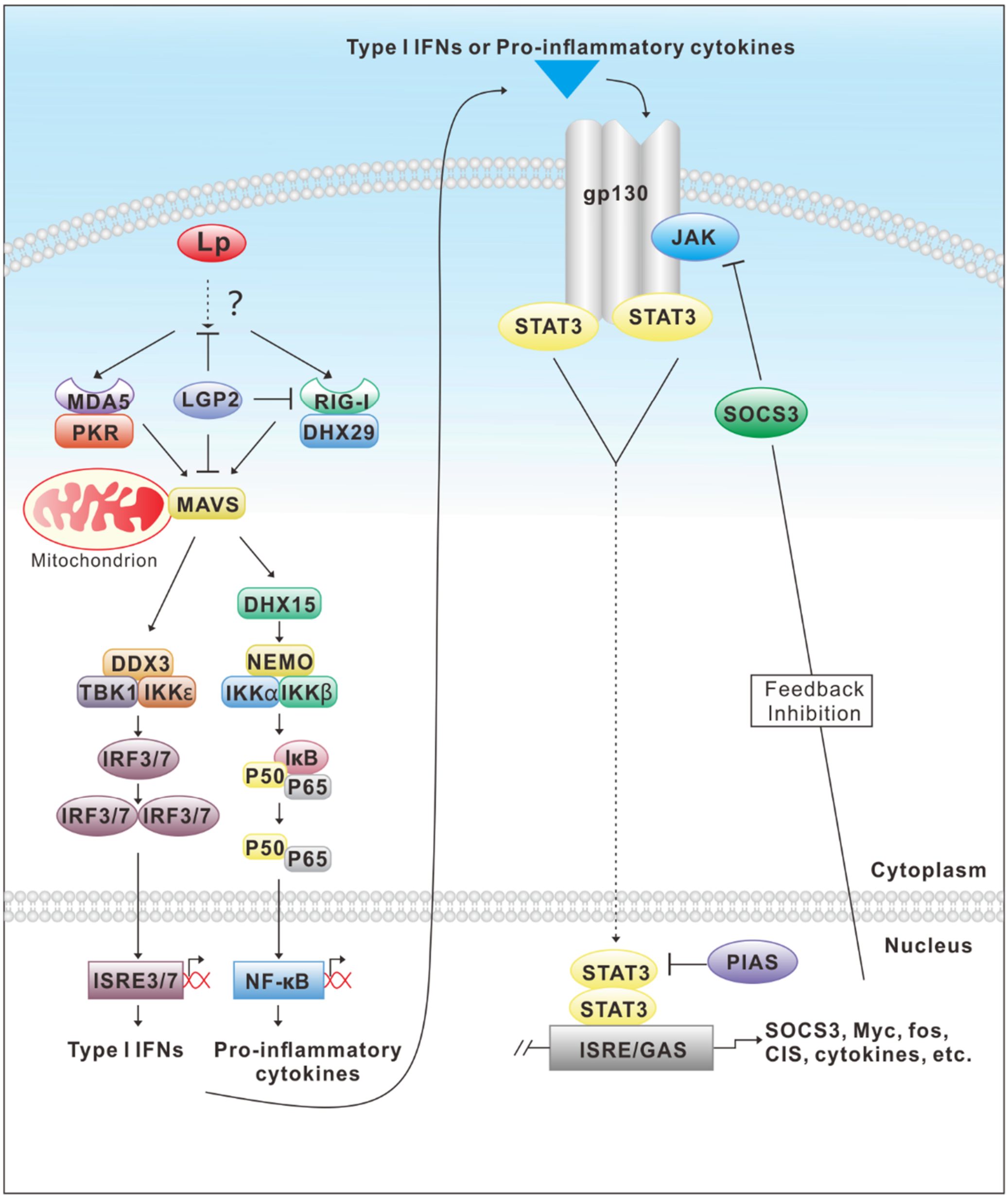

Figure S2. A proposed model for the activation of STAT3 by the LCMV Lp. RdRp activity of Lp may activate both RIG-I and MDA5, thereafter activating IKKε and IRF3/7 to induce the production of IFN-I and NF-κB to induce pro-inflammatory cytokines, respectively. Secreted IFN-I and pro-inflammatory cytokines, such as IL-6 and IL10, then bind their receptors to active the tyrosine kinases of the JAK family and subsequently the STAT3 signaling pathway.
